# Effect of aquatic physical therapy on pain perception, functional capacity and quality of life in older people with knee osteoarthritis: study protocol for a randomized controlled trial

**DOI:** 10.1186/s13063-017-2061-x

**Published:** 2017-07-11

**Authors:** Guilherme Eleutério Alcalde, Ana Carolina Fonseca, Thais Fernanda Bôscoa, Mirella Regina Gonçalves, Gabriele Candido Bernardo, Bruna Pianna, Bianca Ferdin Carnavale, Camila Gimenes, Silvia Regina Barrile, Eduardo Aguilar Arca

**Affiliations:** 1 Universidade do Sagrado Coração, Street Irmã Arminda, 10-50, Jardim Brasil, Bauru, SP Brazil; 2 Universidade Federal de São Carlos, São Carlos, SP Brazil

**Keywords:** Hydrotherapy, Pain, Quality of life, Osteoarthritis, Aged

## Abstract

**Background:**

Aquatic therapy promotes short-term benefits for patients with knee osteoarthritis (OA), and it may be the first therapeutic option for this pathological condition. The objective of this study was to investigate the effects of an aquatic therapy program on pain intensity, functional ability, and quality of life in older people with knee OA.

**Methods/design:**

This is a parallel, two-arm, open, randomized controlled clinical trial with older people with knee OA. Volunteers will be allocated to an aquatic intervention group (WG), subjected to the intervention, or to a control group, not be subjected to any kind of intervention. Data collection pre- and postintervention will be composed of the evaluation of the perception of pain by visual analogue scale with application of nociceptive stimuli in four anatomical points of the knee, functional fitness tests, and application of the World Health Organization Quality of Life Scale abbreviated version and Western Ontario and McMaster Universities Osteoarthritis Index. The program will last 12 weeks, consisting of aerobic and functional exercises in the form of circuit training.

**Discussion:**

The objective of this clinical trial is to evaluate the effect of aquatic therapy in elderly patients with knee OA. The study is guided by practice-based scientific evidence for the use of aquatic rehabilitation exercises. It is expected that the WG volunteers will show reduced pain intensity, increased flexibility, and improved functional capacity and quality of life. It is believed that the desired results can be attributed to physical and physiological effects of immersion in warm water associated with the exercise protocol proposed. The data will be published after completion of the study.

**Trial registration:**

Brazilian Registry of Clinical Trials (ReBEC) registration number: RBR-78h48d. Registered on 19 August 2015.

**Electronic supplementary material:**

The online version of this article (doi:10.1186/s13063-017-2061-x) contains supplementary material, which is available to authorized users.

## Background

Knee osteoarthritis (OA) is a chronic degenerative disease of an inflammatory nature that is characterized by changes in the articular cartilage, the presence of fibrillation areas, and cracking and thickening of the subchondral bone. Clinically, it is associated with pain, stiffness, deformity, and loss of functional capacity [1, 2]. Approximately 10% of the population over the age of 60 years is affected by OA; 80% of this population have movement restrictions, and 25% have functional limitations that compromise the performance of daily activities [3, 4].

Exercise is one of the therapeutic strategies that helps to minimize the deleterious effects on the musculoskeletal system generated by aging while preserving independence; promoting weight control; and improving or maintaining quality of life, functional capacity, and emotional well-being [5, 6]. However, the practice of exercises performed on the ground can aggravate joint pain and increase the risk of falls in this population [7, 8].

Aquatic physiotherapy is considered a safe and effective tool in the treatment of knee OA because immersion in hot water decreases the joint overload and pain symptoms and improves functional capacity and quality of life [9–11]. On the basis of these assumptions, the following hypothesis was formulated: Aquatic physical therapy programs contribute to the increase in the pain threshold, improved functional capacity, and improved quality of life in older adults with knee OA. The objective of the study is to verify the effects of an aquatic physiotherapy program on the perception of pain, functional capacity, and quality of life in older people with knee OA.

## Methods

**Fig. 1:**
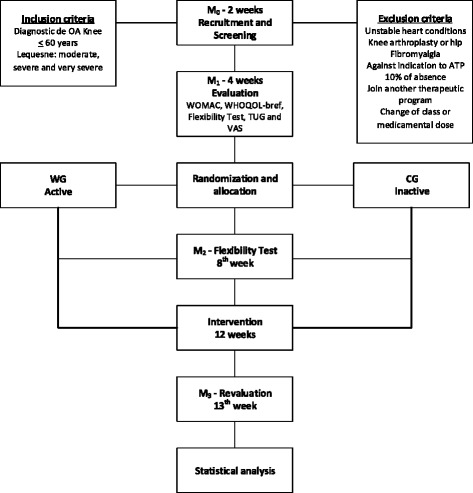
Flow diagram of the randomized clinical trial. Provides detailed information about volunteer recruitment and follow up of 12 weeks during the study. *ATP* Aquatic physiotherapy, *CG* Control group, *M*_*0*_ Moment 0, *M*_*1*_ Moment 1, *M*_*2*_ Moment 2, *M*_*3*_ Moment 3, *OA* Osteoarthritis, *TUG* Timed Up and Go, *VAS* Visual analogue scale, *WG* Aquatic intervention group, *WHOQOL-BREF* World Health Organization Quality of Life Scale abbreviated version, *WOMAC* Western Ontario and McMaster Universities Osteoarthritis Index

**Fig. 2:**
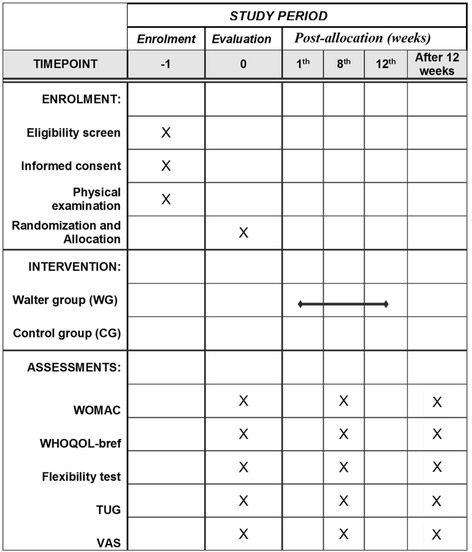
Standard Protocol Items: Recommendations for Interventional Trials (SPIRIT) checklist providing information about volunteer recruitment and variables evaluated in each period according to SPIRIT recommendations. *CG* Control group, *TUG* Timed Up and Go, *VAS* Visual analogue scale, *WG* Aquatic intervention group, *WHOQOL-BREF* World Health Organization Quality of Life Scale abbreviated version, *WOMAC* Western Ontario and McMaster Universities Osteoarthritis Index

The protocol was developed in accordance with the Standard Protocol Items: Recommendations for Interventional Trials (SPIRIT) and Consolidated Standards of Reporting Trials (CONSORT) guidelines and checklists. *See* Additional file 1 for a populated SPIRIT checklist and Fig. 1 for the recommended SPIRIT figure (Fig. 2).

### Overview of research design

This study is a two-arm, parallel, randomized controlled therapeutic clinical trial in which elderly volunteers with knee OA are being recruited.

### Registry of clinical trials

The study protocol was published in the Brazilian Registry of Clinical Trials (registration number RBR-78h48d).

### Availability of data and materials

The evaluations will be conducted at the Research Laboratory for Physical Therapy, and the intervention program will be held at Therapeutic Pools Laboratory of the Universidade do Sagrado Coração, Bauru, São Paulo, Brazil. The researchers will be responsible for the assessment, reassessment, and supervision of the intervention program.

### Inclusion criteria

Participants in the study will be elderly volunteers aged 60 years or older with a diagnosis of knee OA who have been referred by a medical specialist in the City of Bauru, SP, Brazil. For the purposes of inclusion, the precise degree of OA will not be considered, but classification as moderate, serious, or very serious will be undertaken in accordance with the Lequesne Algofunctional Index [12].

### Exclusion criteria

Volunteers will be excluded who present with unstable heart conditions, fibromyalgia, deambulation disability, knee or hip arthroplasty, and contraindications for aquatic physiotherapy [13]. Also excluded will be those who miss two consecutive sessions without justification, as well as volunteers who are absent from 10% of the intervention sessions and who change their dose or class of medication.

### Baseline characteristics

After recruitment and initial screening, details will be collected covering personal and socioeconomic data, lifestyle, medications, identification of the existence of other diseases, anthropometric assessments, and baseline blood pressure [14–16].

### Randomization and allocation

The randomization method used will be a simple draw conducted by a research employee. Volunteers will then be divided into two groups: an aquatic intervention group (WG; *n* = 15) and a control group (CG; *n* = 15) [17]. CG volunteers will not perform any type of exercise or physical therapy.

### Procedures

#### World Health Organization Quality of Life Scale abbreviated version

The World Health Organization Quality of Life Scale abbreviated version (WHOQOL-BREF) is an instrument that has four domains: physical, psychological, social, and environmental. The WHOQOL-BREF comprises 26 questions, 2 of which are centered on the general quality of life, and the remaining 24 of which cover other facets of the instrument [18].

#### Western Ontario and McMaster Universities Osteoarthritis Index

The Western Ontario and McMaster Universities Osteoarthritis Index (WOMAC) is a specific and reliable instrument for individuals with knee OA. This questionnaire consists of three domains: section A - pain (5 questions), section B - joint stiffness (2 questions), and section C - physical function (17 questions). The questions should be answered according to the subject’s perception of pain, joint stiffness, and level of physical function within the last 72 h. The WOMAC scores are presented on a Likert scale in which each question receives a score ranging from 0 to 100, distributed as follows: none = 0, light = 25, moderate = 50, severe = 75, and extreme = 100. The results will be determined from the sum of points of each question and divided by the number of domain questions. In this way, the final three scores will be obtained, one for each domain [19, 20].

### Pain perception assessment

A dolorimeter (Palpeter®; Sunstar Suisse, Etoy, Switzerland), in the form of 1 kg, and a visual analogue scale (VAS) will be used. The dolorimeter consists of a plastic casing with a retractable, cylindrical aluminum ferrule 10 mm in diameter. The examiner should hold the plastic casing with the thumb and third finger by placing the index finger on the instrument orifice. Thus, the pressure exerted will be maintained for 3 seconds on each of the four previously defined anatomical points: (a) lateral joint interline, (b) medial joint interline, (c) medial condyle of the femur, and (d) the lateral condyle of the femur. Then, the volunteers should be classified for pain intensity according to VAS. This consists of a 10-cm line containing numbers ranging from 0 to 10, where 0 represents “no pain” and 10 represents “worst possible pain” [21].

### Timed Up and Go test

The Timed Up and Go (TUG) test protocol comprises the following sequence of movements: rising from a chair, walking 3 m, turning around, and sitting back down in the chair. The time it takes the subject to perform this sequence of movements is recorded [22]. The best time of three attempts is taken into consideration.

### Flexibility test (sit and reach)

Volunteers should be barefoot and sit facing the box with the lower limbs in extension and adduction. The hands should be positioned one on top of the other, and the upper arms should be in the vertical position. The body should be leaned forward, and the fingertips should reach over the graduated scale as far as possible without bending the knees and without the use of swinging movements [23]. The best distance of three attempts will be considered.

### Intervention and follow-up periods

The intervention program will last for 12 weeks and consist of a 40-minute session three times per week with the temperature of the pool water maintained at 33 °C. The program will be held at the circuit training facility and will be based on the protocols described by Lau et al. [24], Hale et al. [25], and Lin et al. [26], as noted in Table 1.

**Table 1:** Description of aquatic therapy program components

Components	Repetitions	Description
Motor coordination and agility	4 × 30 seconds and 30 seconds rest	Gait training in anteroposterior, lateral-lateral, and diagonal in the shallow end of the pool (1 m deep). Then they will go up and down the inside of the pool ladder, alternating legs (two steps).
Flexibility	6 × 10 seconds with 10 seconds rest	Unilateral and alternating stretches of the following muscle groups will be done: triceps, greater pectoral, quadriceps, hamstrings, gastrocnemius, and thigh adductors.
Space perception, time and speed of reaction	3 × 1 minute and 30 seconds rest	Pairs will be formed to throw a ball; subjects should perform this activity by performing side-to-side and anteroposterior movements in the shallow end of the pool.
Balance	4 × 1 minute and 30 seconds rest	Standing position with feet on boards for balance training. Support may be bipedal or single-leg. To increase the degree of difficulty, the eyes may remain closed.
	10 × 10 seconds and 10 seconds rest	Triceps: Aquatubes will be used to perform the exercises. Individuals remain standing in the middle of the pool with raised shoulders and elbows close to the body, conduct extension and flexion, keeping the forearms pronated.
		Greater pectoral: Individuals standing, holding shoulder flexion at 90 degrees, holding the plank of exercises and perform flexion and elbow extension.
Muscle training	6 × 40 repetitions and 10 seconds rest	Quadriceps: While standing in the shallow end of the pool, exercise will be conducted in closed kinetic chain (squat).
	10 × 10 seconds and 10 seconds rest	Rectus abdominals: For performing abdominal exercises, individuals will support their hands on the horizontal bar of the pool and hold the “kicking” movement, putting bilaterally foot on the wall of the pool and then returning to the ground.

Thus, the study will be divided into four moments: M_0_ (recruitment and screening), M_1_ to test and evaluative measures, and M_2_ and M_3_ for evaluation of the flexibility and reevaluation of tests and measurements (Fig. 1).

### Sample size

The sample size calculation was conducted using the G Power program with an alpha of 5% and beta of 80%, and thus we obtained the sample size of 15 volunteers in each group.

### Analysis

The normality of the data will be verified with the Shapiro-Wilk test. Parametric data will expressed as mean and SD, using Student’s *t* test for independent samples to compare the groups and two paired samples to compare the times. For the analysis of repeated measures, analysis of variance with a post hoc Tukey test will be used. Nonparametric data will be expressed as median, maximum, and minimum values. The Mann-Whitney *U* test will be used to compare the groups, and for comparison of moments, the Wilcoxon test will be applied. In all tests, results will be judged to be statistically significant when α values are <0.05.

## Discussion

The objective of this clinical trial is to evaluate the effect of aquatic physical therapy in older people with knee OA. The study is guided by practice-based scientific evidence for the use of aquatic rehabilitation exercises for this condition.

Upon completion of data collection, it is expected that the WG volunteers will benefit from increased flexibility through the Wells test, improved functional capacity through the TUG test, reductions in scores on the WOMAC, and quality of life assessed by the WHOQOL-BREF. In the CG, no changes in the values of any variable analyzed are expected. It is believed that the desired results could be attributed to physical and physiological effects of immersion in heated water associated with the proposed exercise protocol. The data will be published after the study is completed.

### Trial status

Data are currently being collected.

## Additional file

Additional file 1: SPIRIT 2013 checklist: recommended items to address in a clinical trial protocol and related documents. (DOC 120 kb)

## Abbreviations

CG: Control group
CONSORT: Consolidated Standards of Reporting Trials
M_0_: Moment 0
M_1_: Moment 1
M_2_: Moment 2
M_3_: Moment 3
OA: Osteoarthritis
SPIRIT: Standard Protocol Items: Recommendations for Interventional Trials
TUG: Timed Up and Go
VAS: Visual analogue scale
WG: Aquatic intervention group
WHOQOL-BREF: World Health Organization Quality of Life Scale abbreviated version
WOMAC: Western Ontario and McMaster Universities Osteoarthritis Index
